# Supplementary material for: Safety and Immunogenicity of a Stable, Cold-Adapted, Temperature-Sensitive/Conditional Lethal Enterovirus A71 in Monkey Study
Source: Viruses. 2021 Mar 9;13(3):438. doi: 10.3390/v13030438 (PMC8001754; doi:10.3390/v13030438)
Supplement: Supplementary file 1 [file viruses-13-00438-s001.pdf]

Supplementary Information

Table S1: The number of nucleotide (NT), and corresponding amino acid (AA), mutations that occurred in each of the genomic segments of virus strains derived from temperature-sensitivity reversion study. Results are compared with the genome of EV71:TLLβP20. (R): reversion to wild-type.

**Viral Gene Region/Protein**		**TLLβ (37°C -P1)**		**TLLβ (37°C -P2)**		**TLLβ (37°C -P3)**		**TLLβ (37°C -P4)**		**TLLβ (37°C -P5)**		**TLLβ (37°C -P6)**	
		NT	AA	NT	AA	NT	AA	NT	AA	NT	AA	NT	AA
5’-UTR	“Cloverleaf”					1(R)		1(R)		1(R)			
(1-746)	IRES												
P1	VP4												
(747-3332)	VP2												
	VP3												
	VP1									1	1	2	1
P2	2A											1(R)	1(R)
(3333-5066)	2B												
	2C												
P3	3A												
(5067-7325)	3B												
	3C												
	3D							1(R)	1(R)	3(1R)	2(1R)	3(1R)	2(1R)
3’-UTR													
(7326-7411)													
Total						1(R)		2(R)	1(R)	5(2R)	3(1R)	6(2R)	4(2R)

Table S2: The number of nucleotide (NT), and corresponding amino acid (AA), mutations that occurred in each of the genomic segments of EV-A71 present in the serum samples of two monkeys (2889M and 2890M) collected on Day 4 and Day 8 PI. Results are compared with the complete genome of EV71:TLLβP20. (R): reversion to wild-type.

Viral Gene Region/Protein		Monkey (2889M)				Monkey (2890M)			
		Day 4		Day 8		Day 4		Day8	
		NT	AA	NT	AA	NT	AA	NT	AA
5'-UTR	"Cloverleaf"								
(1-746)	IRES								
P1	VP4								
(747-3332)	VP2								
	VP3								
	VP1	1				5	1(R)	6	1(R)
P2	2A	3	2	3	2	3	2	3	2
(3333-5066)	2B								
	2C	2(1R)	1(R)	1		1(R)		1(R)	
P3	3A								
(5067-7325)	3B								
	3C								
	3D	1(R)	1(R)	1(R)	1(R)	1(R)	1(R)	1(R)	1(R)
3'-UTR									
(7326-7411)									
Total		7(3R)	4(2R)	5(1R)	3(1R)	10(2R)	4(2R)	11(2R)	4(2R)

Table S3. Nucleotide and amino acid mutations in TLLβ, TLLβ-P20 and TLLβ-R37P5 compared to parental ST strain. Mutations at conserved sites are highlighted by yellow colour. re: reverted; uc: unchanged.

Genome regions	Nucleotide mutation v.s. ST strain			Amino acid mutation v.s. ST strain		
5'UTR	TLLβ	TLLβ-P20	TLLβ-R37P5	TLLβ	TLLβ-P20	TLLβ-R37P5
	A58G	re	re			
	T69C	re	re			
	T91C	T91C	T91C			
	T591C	T591C	T591C			
	A722G	A722G	A722G			
VP2	G1406A	G1406A	G1406A			
	C1451T	C1451T	C1451T	uc	uc	uc
	G1574T	G1574T	G1574T	uc	uc	uc
	A1703G	A1703G	A1703G	uc	uc	uc
VP3	A1819G	A1819G	A1819G	H358R(VP3-H35R)	H358R	H358R
	C1900A	C1900A	C1900A	A385D (VP3-A62D)	A385D	A385D
VP1	G2481A	G2481A	G2481A	D579N (VP1-D12N)	D579N	D579N
	G2736A	G2736A	G2736A	G664S (VP1-G99S)	G664S	G664S
	C2740T	C2740T	C2740T	T665I (VP1-T100I)	T665I	T665I
	C2749T	C2749T	C2749T	P668L (VP1-P103L)	P668L	P668L
	C2761T	C2761T	C2761T	A672V (VP1-A107V)	A672V	A672V
	A2891T	A2891T	A2891T	L715F (VP1-L150F)	L715F	L715F
	uc	uc	C3112T	uc	uc	A789V
	T3162G	T3162G	T3162G	S806A (VP1-S241A)	S806A	S806A
	G3318A	G3318A	G3318A	A858T (VP1-A293T)	A858T	A858T
2A	C3423T	C3423T	C3423T	L893S (2A-L31S)	L893S	L893S
	T3424C	T3424C	T3424C			
	C3467T	C3467T	C3467T	uc	uc	uc
	T3577C	T3577C	T3577C	I944T (2A-I82T)	I944T	I944T
2B	C3895T	C3895T	C3895T	A1050V (2B-A38V)	A1050V	A1050V
2C	uc	G4619A	G4619A	uc	uc	uc
	T5039C	T5039C	T5039C	uc	uc	uc
3A	T5251Y	re	re	M1502S (3A-M62S)	uc	uc
	G5262A	G5262A	G5262A	A1506T (3A-A66T)	A1506T	A1506T
3C	T5487C	T5487C	T5487C	C1581R (3C-C33R)	C1581R	C1581R
	G5676A	G5676A	G5676A	A1644T (3C-A96T)	A1644T	A1644T
3D	G6155T	G6155T	G6155T	E1803D (3D-E72D)	E1803D	E1803D
	uc	T6591G	uc	uc	W1949G	uc
	uc	uc	C6710T	uc	uc	uc
	uc	uc	G6976A	uc	uc	R2077K
	C7273T	C7273T	C7273T	A2176V (3D-A445V)	A2176V	A2176V
3'UTR	A7400T	A7400T	A7400T			
